# Whole Body Center of Mass Estimation with Portable Sensors: Using the Statically Equivalent Serial Chain and a Kinect

**DOI:** 10.3390/s140916955

**Published:** 2014-09-11

**Authors:** Alejandro González, Mitsuhiro Hayashibe, Vincent Bonnet, Philippe Fraisse

**Affiliations:** 1 INRIA (Institut National de Recherche en Informatique et en Automatique), DEMAR Team, Montpellier 34095, France; E-Mails: gonzalezde@lirmm.fr (A.G.); fraisse@lirmm.fr(P.F.); 2 LIRMM (Laboratoire d'Informatique, de Robotique et de Microélectronique de Montpellier), University of Montpellier 2, Montpellier 34090, France; 3 Movement to Health (M2H) Laboratory, EuroMov, University of Montpellier 1, Montpellier 34090, France; E-Mail: bonnet.vincent@gmail.com

**Keywords:** statically equivalent serial chain (SESC), identification, center of mass (CoM), subject-specificity, Kinect

## Abstract

The trajectory of the whole body center of mass (CoM) is useful as a reliable metric of postural stability. If the evaluation of a subject-specific CoM were available outside of the laboratory environment, it would improve the assessment of the effects of physical rehabilitation. This paper develops a method that enables tracking CoM position using low-cost sensors that can be moved around by a therapist or easily installed inside a patient's home. Here, we compare the accuracy of a personalized CoM estimation using the statically equivalent serial chain (SESC) method and measurements obtained with the Kinect to the case of a SESC obtained with high-end equipment (Vicon). We also compare these estimates to literature-based ones for both sensors. The method was validated with seven able-bodied volunteers for whom the SESC was identified using 40 static postures. The literature-based estimation with Vicon measurements had a average error 24.9 ± 3.7 mm; this error was reduced to 12.8 ± 9.1 mm with the SESC identification. When using Kinect measurements, the literature-based estimate had an error of 118.4 ± 50.0 mm, while the SESC error was 26.6 ± 6.0 mm. The subject-specific SESC estimate using low-cost sensors has an equivalent performance as the literature-based one with high-end sensors. The SESC method can improve CoM estimation of elderly and neurologically impaired subjects by considering variations in their mass distribution.

## 1. Introduction

It is estimated that by the year 2050, 16% of the world's population will be over 65 years old. The steady increase in the population's age will not occur uniformly all over the world. Instead, this percentage is expected to be larger in the developed world, including the USA, Japan and European countries [1]. Such predictions are today driving the demand for tools that can improve quality of life for the elderly population with financially reasonable solutions. For example, robot-based rehabilitation systems and sensors meant to detect and prevent falls are now being created to support unassisted living[2–6] and to maintain high-level motor functions without increasing the cost of human resources.

Human balance is strongly related to the position and velocity of the whole body center of mass (CoM).For instance, the ground projection of the CoM must remain inside the support polygon(the convex hull defined by all supporting contacts[7]) during both quiet standing and functional reach tasks. Therefore, balance can be determined by observing the CoM trajectory. Lafond *et al.* [8] and Jaffrey [9] have described the methods most commonly used for CoM estimation and discussed their applications. The so-called segmentation methods (sometimes also referred to as kinematic methods) are the most widely used, due to the ease of their implementation. The body's CoM is obtained as the weighted sum of the CoM position of each body segments. The weight of each segment's CoM position is the ratio of the segment to total body mass. Currently, the information required for this calculation is taken from tables compiled in anthropometric studies. However, these studies were performed mainly on cadavers or in live, young and fit individuals [10,11] and only represent the population from which they were compiled. These values should be adjusted for age, sex and fitness level [12],in order to minimize the estimation error. In addition, segmentation methods for CoM estimation require tracking the position of individual body segments, and this is usually achieved using marker-based motion capture systems, available only in a laboratory setting. Baker [13] detailed the clinical uses of such motion capture systems and enumerated their faults.

Different methods have been developed to obtain a subject-specific whole body CoM position. For example: in [14], simple planar measurements are used to determine either the mass or the CoM position of a body segment, while the other is obtained from an anthropometric table. Another approach is to approximate body segments as known geometric bodies, e.g., [12]; the accuracy of this method is dependent on the geometric segmentation of the body and the quality of the measurements. Based on the work of Espiau and Boulic [15], Cotton *et al.* [16] proposed the statically equivalent serial chain (SESC) method to determine whole body CoM position and showed how the same model could be used to locate the subject-specific CoM. In short, the SESC method translates the subject's mass distribution to the geometry of a linked chain, and like other segmentation methods, it requires tracking the subject's movements. A personalized SESC can be established after an identification phase, which requires high-end sensors, such as a motion capture device and a force platform. Venture *et al.* [17] presented a real-time estimation of dynamic parameters for human subjects. They contain mass distributions and inertial parameters that can also be used to estimate CoM position and can be identified by measuring ground reaction forces while tracking body segment positions. For this, they require an accurate motion capture system and prior knowledge of the segments' lengths. In addition, the method described in [17,18] requires the subject to move each individual segment with a certain speed; something that may be difficult for some motor-impaired subjects.

Portable tools are necessary to make CoM estimation available in both the clinical settings and the home environment. Stone and Skubik [19], for example, used a Kinect (Microsoft^®^ Corporation, Redmont, WA, USA) to measure step time and length in older adults. They found good agreement with measurements obtained with a Vicon system (Oxford Metrics Group, Oxford, UK). Additionally, Clark *et al.* [20] and Yang *et al.* [21] have evaluated the Kinect's skeletal measurements for postural analysis and CoM determination, respectively. Both of these works found a good correlation between the angular measurements made by the Kinect and a high-end motion capture system. Similarly, several studies analyzed the Wii balance board (WBB by Nintendo^®^ Co. Ltd., Kyoto, Japan) to assess balance [22] and its accuracy during postural tasks [23–25]. Their major findings were the following: the instantaneous estimates of the center of pressure (CoP) location obtained with a WBB and a laboratory grade force platform are fairly similar [23], with an offset difference smaller than 5 mm [24]; the measurements are reliable across trials performed with the same WBB [24]; and the WBB noise makes estimating CoP velocity a difficult task [23,25].

If body kinematics can be obtained in the patient's home, a real-time subject-specific and whole body CoM can be determined and used to assess balance in a customized manner, complementing home-based physical rehabilitation programs [26]. In [27], we reported a preliminary result showing that color and depth (RGB-D) cameras, such as the Kinect, can be used to accurately perform a subject-specific CoM estimation through SESC. The identification phase was performed using CoP information obtained with a WBB, demonstrating that creating a subject-specific SESC with portable, low-cost sensors is possible.

In this work, we investigate the performance of the personalized CoM tracking when compared to a literature-based estimate. We also compare the results of a personalized CoM estimation obtained from portable and high-end sensors. That is, we estimate whole body three-dimensional CoM position with: (i) a SESC identified using a Vicon motion capture system and an AMTI-OR6 (AMTI6 by Advanced Mechanical Technology Inc., Waterton, MA, USA) force-platform; (ii) literature-based parameters [10] and Vicon measurements; (iii) a SESC obtained using the Kinect and WBB;(iv) and literature-based parameters and Kinect's angular measurements. We compare the estimated CoM ground projection for each case to the measured AMTI6 data, which we considered as the ground truth. In this way, we aim to show the performance of the subject-specific CoM estimate using portable sensors and compare its accuracy to the high-end sensor case.

## 2. Methods

In order to determine a personalized CoM position, two SESCs were identified for each subject: one using high-end sensors, the other with low-cost portable ones. In addition, a literature-based estimate was performed using the anthropometric data provided by Winter [10] and kinematic measurements from the Vicon and the Kinect. Thus, wee valuated the performance of the following estimates:

(i) Vicon-SESC, identified from the Vicon-AMTI6 data;
(ii) Vicon-Winter using Vicon's body posture measurements and Winter's anthropometric data;
(iii) Kinect-SESC, identified from data obtained with the Kinect-WBB; and
(iv) Kinect-Winter using Kinect's postural measurements and Winter's anthropometric data.

**Figure 1.**
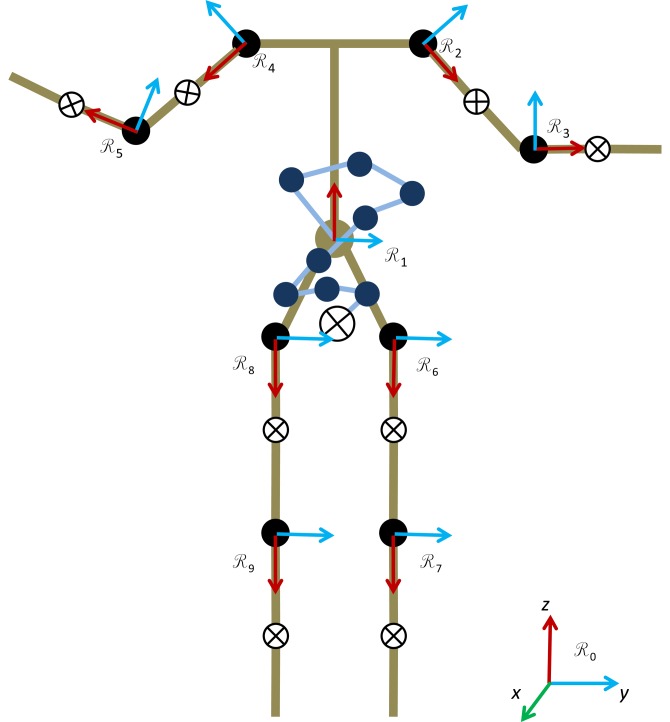
A human-like skeleton model made up of nine rigid body segments. The statically equivalent serial chain (SESC) representing the center of mass (CoM) position is attached to the torso and depicted in blue.

Due to the limited information available from the Kinect, we assume a skeletal model composed of nine rigid bodies (see Figure 1). The hands, feet and head were grouped with their neighboring links, since due to their relative small masses or range of motion (for the head), they have only a small influence on the variation of the whole body CoM position. Details on the SESC method and its identification procedure can be found in [16]. As described in [28], we assume the same mass distribution on the subject's right and left sides. Currently, the identification of SESC parameters requires a subject to perform a series of static postures. To determine if a certain posture can be considered static, we observe CoP displacement and the change in orientation of body segments during a 1 s window. We then compare the standard deviation (std) of the displacements to experimentally set thresholds, and if the displacements were smaller than these thresholds, the window was considered static.

Once the subject-specific SESC models were established, the ground projection of the estimated CoM of the four configurations, *i.e.*, (i)–(iv), were compared to the measured CoP position from the AMTI6 force platform. We considered measured CoP during static postures to be the ground truth of the ground projection of the CoM. To compare the different CoM estimations, we determined: the root mean square error (rmse) between them and the measured CoP, as well as their std for 40 static postures not used for the identification of the SESC models.

### 2.1. Statically Equivalent Serial Chain

For the sake of completeness, we briefly present the SESC method, which can be used to determine a subject-specific CoM. The position of the CoM (*C⃗_M_*) of any multi-body structure can be expressed as the end effect or position of an open-ended serial chain, known as the SESC. The structure of the SESC can be defined by the static and geometric parameters of the original whole body structure [16].

For example, take the linked chain shown in Figure 1. A frame 
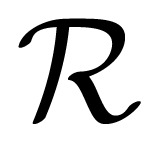
*i* is attached to each link, and the global reference frame is labeled 
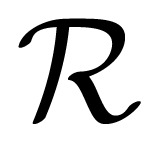
_0_. To simplify calculations, the segment's *z*-axis is aligned with the connecting link. The CoM of a system with an *n* number of links, each with a mass *m*, may be written as:

(1) C→M=1M∑i=1nmi(Ai*ci→+d→0i)

where *^j^d⃗_i_* gives the position of the frame's 
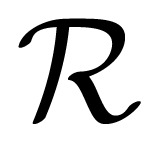
*_i_* origin expressed in frame 
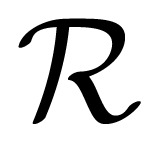
*_j_*, the matrix ${\text{A}}_{i}^{*}$ represents the orientation of 
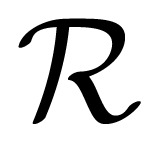
*_i_* with respect to 
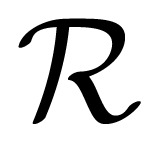
_0_, *c⃗_M_* is the position of the link's CoM in its own reference frame and *M* is the total mass. After expanding, Equation (1) can be rewritten as:

(2) C→M=[I A1*…A9*][0d→1r→1⋮r9→]=[IB][d01→R→]

where **I** is a 3-by-3 identity matrix. The values of *r⃗_i_* contain the mass distribution of the original chain. When all bodies are assumed to be rigid and are connected by means of spherical or hinge joints, vectors *^j^d⃗_i_* are of fixed length and parameters *r⃗*_1…9_ are constant valued vectors and can be explicitly written as:

(3) r1→=1M(c1→m1+d12→(m1+m2)+d14→(m4+m5)+……+d16→(m6+m7)+d18→(m8+m9))

(4) r→2=1M(c→2m2+d23→m3)

(5) $$\vec{r_{3}}=\frac{1}{M}({\vec{c}}_{3}m_{3})$$

(6) $$\vec{r_{4}}=\frac{1}{M}({\vec{c}}_{4}m_{4}{+}^{4}{\vec{d}}_{5}m_{5})$$

(7) $${\vec{r}}_{5}=\frac{1}{M}({\vec{c}}_{5}m_{5})$$

(8) $${\vec{r}}_{6}=\frac{1}{M}({\vec{c}}_{6}m_{6}{+}^{6}{\vec{d}}_{7}m_{7})$$

(9) $${\vec{r}}_{7}=\frac{1}{M}({\vec{c}}_{7}m_{7})$$

(10) $${\vec{r}}_{8}=\frac{1}{M}({\vec{c}}_{8}m_{8}{+}^{8}\vec{d_{9}}m_{9})$$

(11) $${\bar{r}}_{9}=\frac{1}{M}({\vec{c}}_{9}m_{9})$$

In the general three-dimensional case, matrix **B** is of a size of 3-by-3*n*, while *R⃗* is a 3*n*-by-1 vector.

### 2.2. Floating Base

Equation (2) gives the position of the CoM in the global frame. Vector ^0^*d⃗*_1_ is, in general, not a constant, as it depends on the position of the body segment that we consider to be the model's root. When identifying vector *R⃗*, we prefer it to be composed of only constants. This can be achieved by offsetting the CoM position measurements [29]:

(12) $$\begin{array}{cc} {\vec{C}}_{M}{-}^{0}{\vec{d}}_{1} & =B\vec{R}\\ {}^{1}{\vec{C}}_{M} & =B\vec{R} \end{array}$$

We use the torso as the root of the human chain.

### 2.3. Identification

Incomplete or uncertain knowledge of the mass distribution and geometry parameters (*c⃗_j_*, *^i^d⃗_j_* and *m_j_*) prevents us from explicitly writing the parameter vector *R⃗*. In such cases, its value may be experimentally determined by using a set of measurements for which the whole body CoM position and segment orientations are known. In the least squared error sense, the value of vector *R⃗* can be solved by means of the Moore–Penrose pseudo-inverse:

(13) $$\hat{\vec{R}}={\text{B}}^{+1}{\vec{C}}_{M}$$

Using measurements from a large number of postures contributes to refining the estimated parameters, even when not all CoM information is available. Consider the following arrangement for the rows of Equation(2):

(14) [C→1MxC→1MyC→1Mz]=[BxByBz]R→

When a large enough set of *m* stable postures is measured, any combination of rows may be used to create a linear system:

(15) R→ˆ=[Bx,1⋮Bx,mBy,1⋮By,m]+[C1Mx,1⋮C1Mx,mC1My,1⋮C1My,m]=D+[C1Mx,1⋮C1Mx,mC1My,1⋮C1My,m]

For example: when measuring two components of the CoM position, at least (3/2) *n* linearly-independent measurements should be taken. This ensures that D is invertible. Additionally, to decrease the identification error, a large number of measurements for each individual should be used [30], this effectively averages the measurements of each posture and reduces measurement uncertainty.

To estimate a human's SESC parameters in this way, a good CoM measurement should be provided during the initial calibration phase. For this work, we assume that the CoP for static postures, where the acceleration of the CoM is small, offers a suitable estimate of the ground plane projection of the CoM and can be used for the identification. To determine if a certain posture can be considered static, we observe CoP displacement and the change in orientation of body segments during a 1-s window. We then compare the std of the displacements to an experimentally set threshold of 5° for orientations and 3 mm for CoP. If the recorded displacements were smaller than the thresholds, the window was considered static. While CoP does not exactly describe the CoM position, the difference between these two quantities is proportional to the acceleration of the CoM [31]. Additionally, during quiet standing on level ground, the average CoP equals the average ground projection of the CoM [31].We try to minimize the CoP-CoM difference by using at least one second-long static postures.

Finally, care should be taken when interpreting the physical meaning of $\hat{\vec{R}}$. Unless either the mass or the geometry of the objects are known, it is not possible to separate a segment's *c⃗_j_*, *^i^d⃗_j_* or *m_j_* from *r⃗_j_*.

### 2.4. Quality of Identification

Equation (2) presents the CoM position as a linear system of the form:

(16) $$\vec{Y}=\text{W}\vec{\text{X}}+\vec{\rho }$$

where *Y⃗* is a vector of measurements, W is known as a configuration matrix and is of size *r*-by-*c* and *ρ⃗* represents a noise vector. The least squares estimate of vector *X⃗* is such that the Euclidean norm of $\left(\vec{Y}-\text{W}\hat{\vec{X}}\right)$ is minimized.

Mooring *et al.* [32] showed why the condition number of the configuration matrix can be used as a reliable indicator of the quality of the overall identification: a large condition number value indicates a singularity in the configuration matrix, denoting a large numerical sensitivity to data errors or measurement noise. In the Euclidean norm, the condition number of a matrix (17) is defined as the quotient of the largest and smallest singular values (σ). It can be obtained as:

(17) $$\text{cond(W)=}\frac{\sigma_{\max}}{\sigma_{\min}}$$

It is also possible to examine the quality of the individual identified parameters. For this, Khalil and Dombre [30] make use of the parameter relative standard deviation:

(18) $$\sigma_{\hat{X}}{}_{{}_{jr}}\%=100\frac{\sigma_{{\hat{X}}_{j}}}{\left|{\hat{X}}_{j}\right|}$$

where:

(19) $$\sigma_{\rho }^{2}=\frac{{∥\vec{Y}-\text{W}\hat{\vec{X}}∥}^{2}}{r-c}$$

(20) $$C_{\hat{X}}=\sigma_{\rho }^{2}{\left({\text{W}}^{T}\text{W}\right)}^{-1}$$

(21) $$\sigma_{{\hat{X}}_{j}}=\sqrt{C_{\hat{X}}(j,j)}$$

$\sigma_{\rho }^{2}$ is an estimator of the std of the identification error, *C_X̂_* represents the variance-covariance matrix of the estimation parameters and *σ_X̂j_* is the std of the *j*-th parameter. The values of *σ_X̂jr_* % should be observed relative to each other.

For simplicity, let us define *k_j_* as the ratio of the *j*-th parameter relative standard deviation and the smallest one:

(22) $$k_{j}=\frac{\sigma_{{\hat{X}}_{jr}\%}}{\min(\sigma_{{\hat{X}}_{jr}}\%)}$$

If the largest parameter relative standard deviation is more than ten times the smallest one (*k_j_* > 10), the parameter corresponding to the larger value can be assumed to be poorly identified.

### 2.5. Model Reduction

To improve the numerical stability of the configuration matrix, we can reduce the number of unknown values of *R⃗*. For this, employto the main assumptions:

(a) A segment's CoM is restricted to a line that joins two links [10]. For the arm and legs, two of the three components of *r⃗_i_* are known to be zero-valued. The corresponding rows of *R⃗* and columns of D can then be removed.
(b) The human body is bilaterally symmetric, which means that the mass distribution between the body's right and left sides is identical. It is thus possible to reduce the number *r⃗* of parameters needed to describe *C⃗_M_*.

These assumptions allow us to represent *R⃗* as a 7-by-1 vector.

## 3. Volunteers and Experimental Protocol

Data were collected from seven able-bodied male volunteers (age: 29.4 ± 6.6 years; mass: 76.1 ± 16.6 kg; and height: 1.76 ± 0.07 m). Written consent was obtained after explaining the experimental procedure.

**Figure 2.**
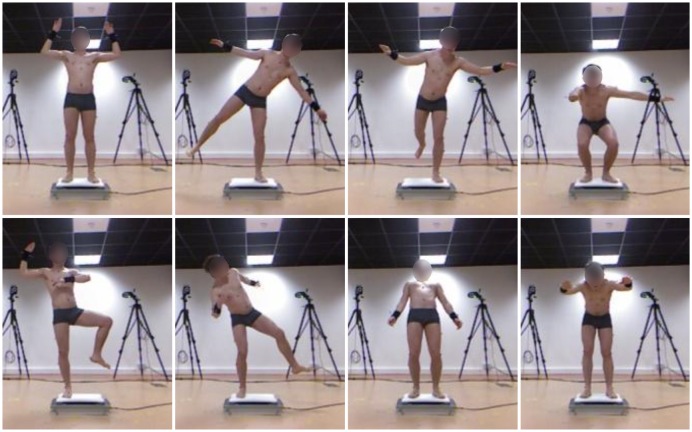
Synchronous recording of Vicon-AMTI-OR6 (AMTI6 by Advanced Mechanical Technology, Inc.) and Kinect-Wii balance board (WBB by Nintendo^®^ Company) measurements. Measuring the same postures with both sets of sensors allows the comparison of SESC-based CoM estimations. Please refer to the Supplementary Material for complete list of postures.

Reflective markers were positioned on the subject following Vicon's Plug-In-Gait model [33]. The volunteers were, in turn, instructed to stand on top of the force-platform and hold 40 static postures, each lasting 5 s. The postures were given to the volunteers by the examiner. Once one was achieved, the volunteer was asked to remain still in order to reduce the accelerations of his CoM. Figure 2 shows some of the postures that were performed. In order to correctly establish the SESC parameter vector, a minimum of 11 linearly-independent postures are required. During this experiment, 40 postures were collected from each subject. This was done to make sure that the orientation of each segment was changed; in this way, the data was considered varied enough so that the parameters corresponding to each segment could be identified. Please refer to the Supplementary Material for more information regarding the calibration postures.

To study the performance of a subject-specific SESC, we simultaneously measured: (a) the orientation of the subject's body segments using an eight-camera Vicon system and a Kinect; and (b) the CoM ground projection with the AMTI6 force platform and the WBB.

### 3.1. Data Acquisition and Processing

Following the standard recommendations, the Kinect was placed facing the subject. The distance to the subject ranged between 2.5 and 3 m. The WBB was placed on top of the AMTI6 force platform to obtain measurement from both devices at the same time, as was previously done by Huurnink *et al.* [23]. Like they did, we found a good agreement between measured CoP trajectories; suggesting that the placement had a minimal effect on the measurement.

The Vicon and AMTI6 data were recorded using Vicon's Nexus software (Oxford Metrics Group, Oxford, UK), thus ensuring synchronization; they were sampled at 100 Hz and 1000 Hz respectively. A custom program recorded the Kinect's skeleton measurements using *OpenNI* (PrimeSense Ltd., Tel Aviv, Israel) and the WBB ones thanks to the *wiiuse*-project [34]. While the nominal sampling frequency of the Kinect is stated to be 30 Hz, it has been found to be irregular [20]. Because of this, the Kinect and WBB data were time stamped. We have found the mean sampling frequency of our measuring system to be about 24 Hz.

All data was then resampled at 15 Hz and low-pass filtered using a zero-phase [35] second-order Butterworth filter with a cut-off frequency of 5 Hz. Finally, the Vicon's Plug-in-Gait model was simplified to have the same segments as the Kinect's skeleton [36].

## 4. Results

For each subject, Table 1 shows the identified $\vec{\hat{R}}$ vector normalized by height, its corresponding parameter relative standard deviations and the condition number of the configuration matrix. These two metrics give us high confidence on the parameters (for details, see [30,32]) Overall, we observe small condition numbers, indicating the good numerical stability of the solution. The parameter relative standard deviations obtained are small and grouped, with the larger values corresponding to the arm and forearm parameters. Additionally, a *t*-test was performed on the identified parameters to determine their significance to the model. Only a few parameters failed the test with *p* > 0.01; these parameters have been highlighted in Table 1.

To show the subject-specificity of the method, we reproduce between parentheses the equivalent SESC parameters as obtained from the literature. To ease the comparison, they are also shown normalized by height. In addition, Table 1 gives subject-relevant information: height, weightandage.

**Figure 3.**
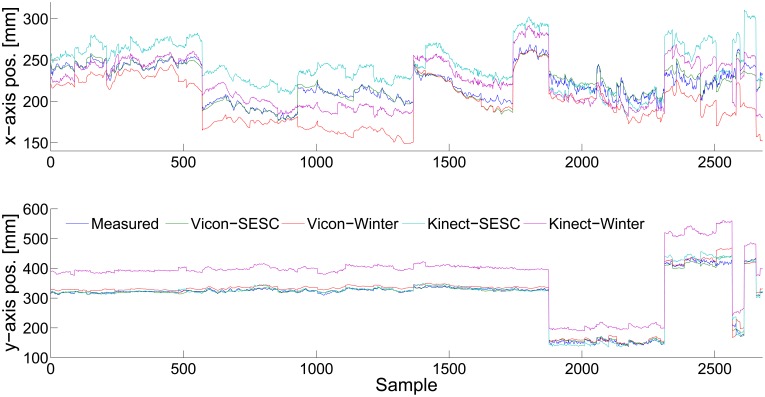
Estimation results for Sb03's. Measured CoP from the AMTI6 platform is shown as a dark blue line. CoM estimation using Vicon and Kinect SESCs are shown in green and light blue lines, respectively. The estimations performed using an anthropometric table [10] with Vicon and Kinect are shown in red and violet, respectively.

Figure 3 shows a typical result for CoM estimation of the validation posture set. Four curves are shown, corresponding to the values obtained using:(i) Vicon-SESC (green line); (ii) Vicon-Winter (red line); (iii) Kinect-SESC (light blue line); and (iv) Kinect-Winter (violet line) estimations. The measured CoP from the AMTI6 force platform is shown with a solid blue line.

**Figure 4.**
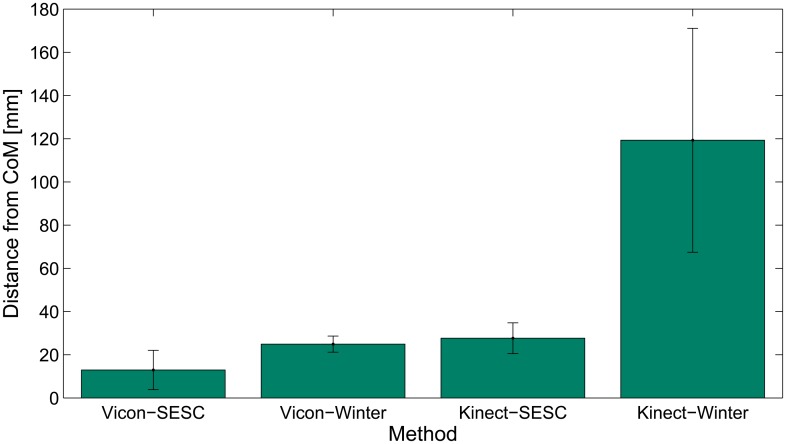
Summary of the performance of each CoM estimation method. The bars correspond to the rmse ± std averaged for all seven subjects. We observe an increase in the accuracy of the identified SESCs with respect to the literature estimates [10]. In addition, the performance of the Kinect-SESC was found to be equivalent to that of the literature-based estimate using high-end sensors.

Figure 4 shows the averaged rmse of the estimation for all seven subjects and all postures. The best performance was the (i) case with a 1289 ± 911 mm average error. The (ii) and (iii) cases performed similarly to each other. The largest estimation error by far came from case (iv), *i.e.*, using literature values with Kinect measurements.

Finally, Table 2 shows the mean and std of the rmse for the (i)–(iv) estimations. This table also shows the error in the subjects' anterior-posterior (AP) and medio-lateral (ML) directions and the coefficient of determination of each case compared to the CoP measurements of the validation posture set.

## 5. Discussion

**Figure 5.**
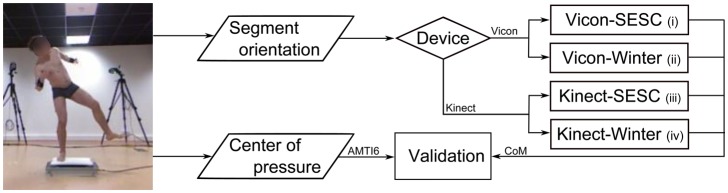
Using the same static postures, the volunteer's CoM is estimated from laboratory-grade measurements with: (i) an SESC and (ii) anthropometric values. To validate the use of low-cost sensors the CoM, estimation is repeated using (iii) an SESC and (iv) anthropometric values.

In this work, we proposed a new method for estimating the whole body center of mass that can be used outside of the laboratory by utilizing the statically-equivalent serial chain and a Kinect. We evaluated the differences between the SESC's CoM estimate obtained fromVicon-AMTI6 data to one created using the Kinect-WBB. For this purpose, the SESC parameters were identified twice over the same static postures, using low-cost and high-end equipment. For completeness, the CoM was also estimated using anthropometric table data [10] to compare these results to the SESC estimate. Figure 5 summarizes this process. Over all, we observed a good performance of the CoM estimations, especially for theVicon-SESC case.

The validation of human CoM estimation methods is an open problem, as this quantity cannot be directly measured. The segmentation method, using laboratory instruments and anthropometric tables, is considered as the standard for whole body CoM estimation [8]. However, no study to our knowledge has yet investigated the possibility of using low-cost instruments to provide a subject-specific CoM estimation. We evaluated the accuracy of portable sensors (the Kinect and the WBB) to estimate CoM by comparing it with that obtained with conventional sensors (Vicon and an AMTI6 force platform). With the Vicon system, the estimation error of the literature-based CoM estimate was found to be 24.9 ± 3.7 mm; this error was reduced to 12.8 ± 9.1 mm using the SESC method. With the Kinect, the literature-based estimate had an error of 118.4 ± 50.0 mm, while the subject-specific SESC error was 26.6 ± 6.0 mm. We find that the subject-specific SESC estimation withlow-cost sensors performed as well as a literature based one with high-end sensors.

We have obtained a high confidence on the identified SESCs. Ideally, the condition number of the configuration matrix would be a small one, close to one (see Table 1), and the ratio of the largest and smallest values of the parameter relative standard deviations (*k*) should not be larger than 10. Nonetheless, a large *k* can be obtained if: (a) the parameter cannot be correctly identified due to noise or (b) the parameter is small, having almost no influence on the CoM. Additionally, we make use of the *t*-test to determine whether or not a parameter's influence is significant for the model. For example: the large *k* values generally found for the *r*_3_ parameter (representing the forearms) indicate that it is difficult to identify, probably due to its small weight. The opposite can be said for *r⃗*_1_ (the torso), which presents small *k* and a *p* < 001, indicating significance. Note that, in general, *r*_3_ is two orders of magnitude smaller than *r⃗*_1_. This is likely due to the relative segments' mass, *i.e.*, the heavy torso influences total CoM more than the light forearms. In addition, the role of the torso link in the CoM estimation is twofold, as it is also the root segment of our model. Finally, a small variation on the identified parameter values between subjects was expected.

Regarding CoM estimation accuracy, we improve on the literature due to the SESC's subject-specific nature. Figure 4 focuses on this. A lower rmse was observed with the Vicon-SESC method than the literature sources [10]. Similarly, using literature values with the Kinect (iv) results in large estimation errors (see Table 2). The SESC estimate for the Vicon-SESC has the same error magnitude for both the AP and ML directions, whereas the Kinect-SESC has a larger mean error on the AP (depth) direction than the MLone. This might be due to the noisy joint positions given by the Kinects keleton, as only one camera is available to reconstruct the kinematics. In contrast, the Vicon skeleton offers better-defined joint obtained from the markers' positions. Finally, the performance of the Kinect-SESC estimate approaches the Kinect's known measurement error [37]. The Vicon-SESC estimate error is larger than that of the Vicon's measurement. This is probably due to the simplified skeletal model.

A two-way ANOVA test was performed to determine the influence of the sensor (Viconor Kinect) and of the origin of the parameters (Winter or SESC) on the rmse. Asignificant effect was found from both factors (*p* < 001). Additionally, a strong interaction of both factors was found (*p* < 001), suggesting that the low rmse found for the Vicon-SESC case was due to both the SESC method and the high-quality measurements of the Vicon system.

## 6. Conclusion

This study sought to determine the accuracy of CoM estimation using the Kinect as the only sensor. The estimation errors are comparable to those obtained using literature-based data from an anthropometric table and a high-end motion capture system. The Kinect-SESC has the advantage of using an inexpensive sensor and does not require lengthy preparations, such as marker placement. In contrast, it requires the subject to perform the SESC's identification procedure. The parameters obtained in this way are constant and subject-specific, so once obtained they can be stored and used again for the same subject unless his mass distribution changes (e.g., weight gains or losses). In addition, since the SESC method offers a kinematic-based estimation of the CoM position, it is also valid for fast, dynamic motions. The static postures (and force platform) are only necessary during the identification phase.

A flexible identification procedure and a portable system could make CoM estimation feasible for the clinical or home environment. This would be helpful for assessing balance training and for non-intrusive patient monitoring [6], while taking into account subject-specificity. The average identification during this experiment required eight minutes of postural recordings. Some of these postures may be too physically demanding for certain patients. To address this, we have also studied the SESC identification for individuals with restricted mobility [38] and considered multiple support surfaces where CoM can be estimated within the patient's range of motion. This method requires only a variety of postures covering the patient's range of available motion. In this way, the SESC method would be also applied to motor-impaired patients.

## Supplementary Material



## Figures and Tables

**Table 1.** Subject information and SESC identified parameters using the Vicon and Kinect data. The parameter vector $\hat{\vec{R}}$ is normalized to subject height to show the differences with literature values. We show the ratio of the parameter relative standard deviations (*κ*), as well as the condition number of the configuration matrix to evaluate the quality of the identification [30]. The highlighted values presented a *p* > 001 during a *t*-test, indicating that they were not significant to the model.

		**Sb01**		**Sb02**		**Sb03**		**Sb04**		**Sb05**		**Sb06**		**Sb07**	
Height (m)		1.78		1.75		1.93		1.72		1.70		1.72		1.75	
Mass (kg)		65.7		76.5		115.1		73.2		55.7		71.4		75.4	
Age (y)		28		29		27		36		25		32		29	
BMI		20.7		24.8		30.9		24.7		19.3		24.1		24.6	
Parameter	**Literature**	**Sb01**		**Sb02**		**Sb03**		**Sb04**		**Sb05**		**Sb06**		**Sb07**	
							
	$\hat{\vec{R}}$	$\hat{\vec{R}}$	*κ*	$\hat{\vec{R}}$	*κ*	$\hat{\vec{R}}$	*κ*	$\hat{\vec{R}}$	*κ*	$\hat{\vec{R}}$	*κ*	$\hat{\vec{R}}$	*κ*	$\hat{\vec{R}}$	*κ*

Vicon-SESC identification															
	(0.0000)	−0.0117	5.14	−0.0100	3.22	0.0122	3.53	0.0132	5.44	0.0079	6.99	0.0133	1.85	−0.0113	4.38
*r⃗*_1_	(0.0000)	0.0021	16.59	0.0003	64.56	0.0040	5.15	0.0047	6.50	0.0022	10.40	0.0052	2.66	0.0165	1.43
	(0.1342)	0.1251	1.00	0.1190	1.00	0.0963	1.00	0.0923	1.00	0.1256	1.00	0.0990	1.00	0.0738	1.02
*r*_2_	(0.0063)	0.0084	7.69	0.0088	5.40	0.0074	3.70	0.0066	8.19	0.0086	5.09	0.0058	4.87	0.0149	2.83
*r*_3_	(0.0022)	0.0036	17.55	0.0011	34.03	0.0000	14922	0.0037	17.75	−0.0007	46.70	0.0020	13.80	−0.0052	7.40
*r*_6_	(0.0256)	0.0249	2.86	0.0246	1.49	0.0255	1.80	0.0307	1.84	0.0234	3.72	0.0266	1.13	0.0447	1.00
*r*_7_	(0.0085)	0.0072	10.72	0.0009	56.62	0.0052	10.75	0.0013	57.98	0.0062	15.37	−0.0100	7.85	0.0066	8.04
cond(**D**)		6.17		9.04		8.32		5.90		8.75		13.02		5.44	

Kinect-SESC identification															
	(0.0000)	0.1042	1.00	−0.1099	1.00	−0.0116	4.70	0.0014	38.23	0.0549	1.00	0.0735	1.00	−0.0275	2.28
*r⃗*_1_	(0.0000)	−0.0075	8.77	0.0167	3.61	0.0319	1.00	0.0354	1.00	0.0309	1.24	0.0282	2.04	0.0442	1.07
	(0.1342)	0.0782	3.00	0.1056	3.10	0.0975	1.50	0.0969	1.13	0.0853	1.70	0.1892	1.71	0.0662	2.43
*r*_2_	(0.0063)	0.0090	17.10	0.0113	13.14	0.0054	7.86	0.0006	112.33	0.0089	6.93	0.0033	38.07	0.0140	7.37
*r*_3_	(0.0022)	−0.0101	12.92	0.0061	24.23	−0.0004	120.21	0.0046	16.79	−0.0042	12.63	0.0068	16.60	−0.0065	12.37
*r*_6_	(0.0256)	0.0452	2.64	0.0391	2.49	0.0308	2.28	0.0288	2.10	0.0420	2.52	0.0496	2.42	0.0654	1.00
*r*_7_	(0.0085)	0.0214	5.78	0.0247	5.81	0.0143	4.92	0.0184	3.87	0.0374	3.06	0.0692	3.35	0.0274	3.03
cond(**D**)		6.88		9.13		7.95		5.12		8.27		9.54		5.96	

BMI: *BMI* = *mass*/(*height*)^2^; Literature refers to the model's SESC parameters assuming literature values [10]; All $\hat{\vec{R}}$ vectors have been normalized by each subject's height.

**Table 2.** The root mean square error (rmse) for each CoM estimation method, in cross-validation for all seven subjects. The error is measured in the world reference frame, averaged for all subjects ± std. anterior-posterior (AP) and medio-lateral (ML) give the anterior-posterior and medio-lateral direction errors, respectively. *R*^2^ is the coefficient of determination of the cross-validation set.

**CoM Estimation**	**Error (mm)**	**AP (mm)**	**ML (mm)**	**R^2^**
Vicon-SESC	12.8 ± 9.1	10.40 ± 6.6	10.2 ± 6.9	0.9 ± 0.1
Vicon-Winter	24.9 ± 3.7	23.16 ± 5.9	13.9 ± 7.3	0.8 ± 0.1
Kinect-SESC	26.6 ± 6.0	23.37 ± 6.8	17.1 ± 8.0	0.8 ± 0.2
Kinect-Winter	118.4 ± 50.0	94.82 ± 70.2	51.8 ± 24.2	−2.8 ± 6.9
