# Supplementary material for: Whole Body Center of Mass Estimation with Portable Sensors: Using the Statically Equivalent Serial Chain and a Kinect
Source: Sensors (Basel). 2014 Sep 11;14(9):16955–71. doi: 10.3390/s140916955 (PMC4208208; doi:10.3390/s140916955)
Supplement: Supplementary file 1 [file sensors-14-16955-s001.pdf]

OPEN ACCESS
sensors
ISSN 1424-8220
www.mdpi.com/journal/sensors

Supplementary Information

Whole Body Center of Mass Estimation with Portable Sensors: Using the Statically Equivalent Serial Chain and a Kinect. *Sensors* 2014, *14*, 16955–16971

Alejandro González [1,2], Mitsuhiro Hayashibe [1,2]*, Vincent Bonnet [3] and Philippe Fraisse [1,2]

[1] INRIA (Institut National de Recherche en Informatique et en Automatique), DEMAR Team, Montpellier 34095, France;
E-Mails: gonzalezde@lirmm.fr (A.G.); fraisse@lirmm.fr (P.F.)

[2] LIRMM (Laboratoire d'Informatique, de Robotique et de Microélectronique de Montpellier), University of Montpellier 2, Montpellier 34090, France

[3] Movement to Health (M2H) Laboratory, EuroMov, University of Montpellier 1, Montpellier 34090, France; E-Mail: bonnet.vincent@gmail.com

* Author to whom correspondence should be addressed; E-Mail: hayashibe@lirmm.fr.

---

1. Identification Postures

The identification of the statically equivalent serial chain (SESC) was done by measuring the orientation of the body segments and center of pressure (CoP) position for 40 static postures. Figures S1–S3 show Sb06 performing the identification procedure. The figures are arranged in chronological order, from left to right. The identification procedure was conceived of as a game-like procedure, and each posture was a slight modification form the previous one. For convenience, the arms were the first segments to be moved; but the poses could have been performed in any order. Moreover, while the poses were assigned to the subject, his ability to accurately produce the posture was not evaluated.

The overall goal during the parameter identification is to obtain postures that cover the whole range of available motions that can be made comfortably by the subject; since the larger the variation in the recorded orientation of a limb is, the smaller its parameter covariance ($C_{\hat{X}}$) will be. If this method is applied to a motor-disabled subject, only the postures he is capable of preforming should be covered; in that case, it is not necessary to use the postures shown in Figures S1–S3. Additionally, a large enough number of linearly-independent measurements should be performed to improve the model identification. For the model used in this paper (composed of seven parameters), an absolute minimum of 11 poses is required.

Furthermore, the set of postures presented here was not designed to optimize the identification. To obtain an optimum, a study of the effects of each posture on the condition number of $\mathbf{W}$ and on $\mathbf{C}_{\hat{X}}$ should be performed. Such a study should take into consideration the subject's range of motion.

Figure S1. Forty static poses used for the identification of a SESC chain.

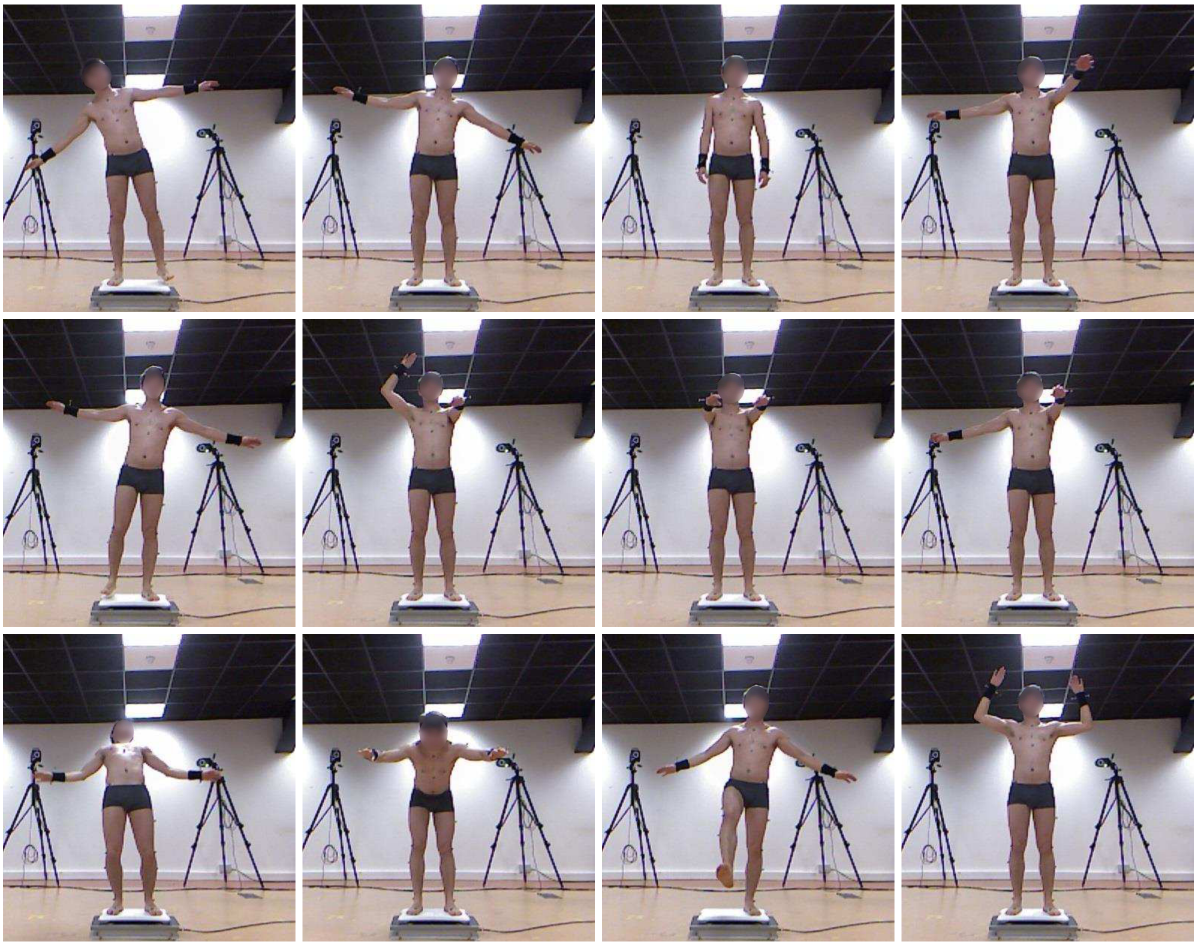

Figure S2. Continued: Forty static poses used for the identification of a SESC chain.

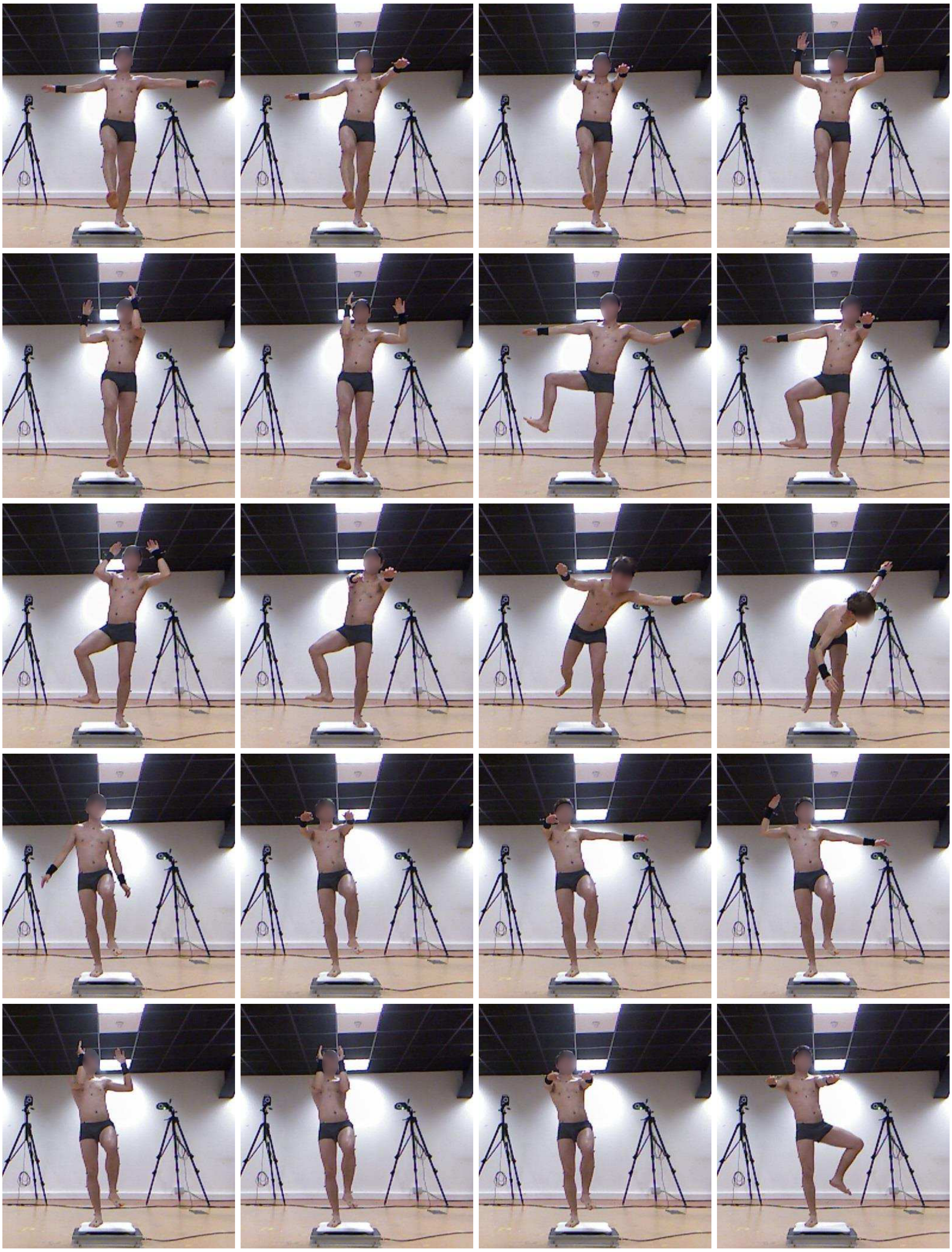

Figure S3. Continued: Forty static poses used for the identification of a SESC chain.

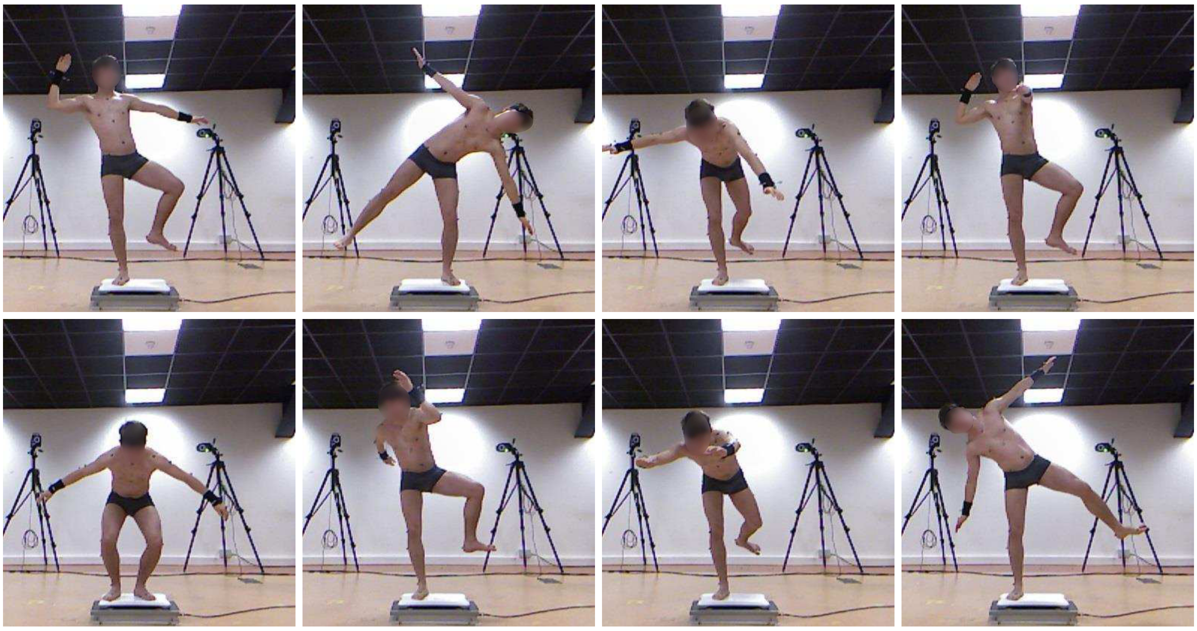

© 2014 by the authors; licensee MDPI, Basel, Switzerland. This article is an open access article distributed under the terms and conditions of the Creative Commons Attribution license (http://creativecommons.org/licenses/by/3.0/).
